# Mouse APOBEC1 cytidine deaminase can induce somatic mutations in chromosomal DNA

**DOI:** 10.1186/s12864-019-6216-x

**Published:** 2019-11-14

**Authors:** Vincent Caval, Wenjuan Jiao, Noémie Berry, Pierre Khalfi, Emmanuelle Pitré, Valérie Thiers, Jean-Pierre Vartanian, Simon Wain-Hobson, Rodolphe Suspène

**Affiliations:** 1 Molecular Retrovirology Unit, Institut Pasteur, CNRS UMR 3569, 28 rue du Dr. Roux, 75724 Paris cedex 15, France; 2 Université Paris Diderot, Sorbonne Paris Cité, Paris, France; 3 Sorbonne Université, Complexité du Vivant, ED515, 75005 Paris, France

**Keywords:** APOBEC1, Cytidine deaminase, Somatic mutations, Nuclear DNA, Cancer

## Abstract

**Background:**

APOBEC1 (A1) enzymes are cytidine deaminases involved in RNA editing. In addition to this activity, a few A1 enzymes have been shown to be active on single stranded DNA. As two human ssDNA cytidine deaminases APOBEC3A (A3A), APOBEC3B (A3B) and related enzymes across the spectrum of placental mammals have been shown to introduce somatic mutations into nuclear DNA of cancer genomes, we explored the mutagenic threat of A1 cytidine deaminases to chromosomal DNA.

**Results:**

Molecular cloning and expression of various A1 enzymes reveal that the cow, pig, dog, rabbit and mouse A1 have an intracellular ssDNA substrate specificity. However, among all the enzymes studied, mouse A1 appears to be singular, being able to introduce somatic mutations into nuclear DNA with a clear 5’TpC editing context, and to deaminate 5-methylcytidine substituted DNA which are characteristic features of the cancer related mammalian A3A and A3B enzymes. However, mouse A1 activity fails to elicit formation of double stranded DNA breaks, suggesting that mouse A1 possess an attenuated nuclear DNA mutator phenotype reminiscent of human A3B.

**Conclusions:**

At an experimental level mouse APOBEC1 is remarkable among 12 mammalian A1 enzymes in that it represents a source of somatic mutations in mouse genome, potentially fueling oncogenesis. While the order *Rodentia* is bereft of A3A and A3B like enzymes it seems that APOBEC1 may well substitute for it, albeit remaining much less active. This modifies the paradigm that APOBEC3 and AID enzymes are the sole endogenous mutator enzymes giving rise to off-target editing of mammalian genomes.

## Background

Apolipoprotein B mRNA editing enzyme catalytic subunit 1, APOBEC1 (A1), is a polynucleotide cytidine deaminase mediating the conversion of cytidine to uridine in RNA. This enzyme was initially described as part of an RNA editing complex involved in the deamination of apolipoprotein B transcript, leading to the production of ApoB48, a triglyceride carrier, from the mRNA encoding ApoB100, a cholesterol carrier [1–3]. This activity, central to lipid metabolism, is restricted to gastrointestinal tissues and requires the APOBEC1 complementation factor ACF for precise targeting of ApoB mRNA [4, 5]. Off-target editing of ApoB mRNA and other mRNAs is also known [6–9]. In addition to this RNA editing activity, A1 enzymes from some species have been shown to act as DNA mutators in vitro [10] as well as on bacterial DNA [11] and even to restrict some retroviruses [12–15], DNA viruses [16–18] and retroelements [19–21] functions otherwise physiologically performed by APOBEC3 family cytidine deaminases.

The *APOBEC3* (*A3*) locus, delineated by two conserved genes, *chromobox 6* and *7* (*CBX6* and *CBX7*), is present in all placental mammals and encodes a diverse repertoire of single stranded DNA cytidine deaminases [22–24]. These enzymes are involved in the restriction of many retroviruses [25–28], DNA viruses [29–31], as well as endogenous retroelements and retrotransposons [32–34]. As a consequence of extensive gene duplications and functionalization in the context of a virus-host arms race the A3 locus is extremely variable among mammals [23, 24, 35, 36]. Phylogenetically, A3 enzymes are made up of three related, but distinct zinc coordination domains referred to as Z1, Z2 and Z3 that can be traced back to the genome of the last common ancestor of placental mammals [24, 36]. It has recently emerged that two human A3 cytidine deaminases, APOBEC3A (A3A) and APOBEC3B (A3B) are capable of introducing numerous somatic mutations in genomic DNA. These observations are supported by experimental data [37, 38] and a posteriori analyses of many cancer genomes, displaying far more mutations and rearrangements than hitherto imagined, where the CG ➔ TA transitions appear to be the dominant mutations [39–41].

Discussion still persists regarding the relative contribution of A3A and A3B enzymes to oncogenesis. A3A is certainly the more active of the two in experimental settings as judged by the genesis of point mutations and double stranded DNA breaks (DSBs) [38, 42–44]. Moreover, cancers can emerge on a *A3B*^*−/−*^ background at a slightly greater frequency [45–47] and cancer genomes analysis reveal 2× more mutations with the A3A specific signature (YTCA) over A3B specific mutations (RTCA) [48–50]. Interestingly, this strong mutagenic feature of A3A has been conserved among most placental mammals, with many A3A related A3Z1 cytidine deaminases demonstrated to elicit nuclear DNA editing and DNA damage [51–53], indicating that the role of those enzymes in innate immunity and DNA catabolism [54, 55] far exceeds the mutagenic threat to self-DNA in evolutionary terms.

Despite this, a few mammals such as opossums, pigs, cats and the entire rodent order have lost the *A3Z1* gene during evolution [23, 24]. However, these animals develop cancer, with notable examples being vaccine associated feline fibrosarcoma and murine lymphoma. Although the sources of mutations driving oncogenesis can be many, the aim of the study was to explore the contribution of APOBEC1 cytidine deaminase to the large number of point mutations and rearrangements evidenced in many cancer genomes. Three lines of evidence suggest APOBEC1 enzymes as a possible candidate. Firstly, the afore mentioned DNA substrate specificity for some mammalian A1 enzymes. Secondly, mouse A1 has recently been shown to exhibit in vitro 5-methylcytidine deaminase activity [56], which is a hallmark of nuclear DNA editing enzymes such as A3A and A3B [38, 57]. Finally, transgenic mice and rabbits engineered to express rabbit *A1* under a hepatotropic promoter developed hepatocellular carcinomas [58]. In the present study, twelve mammalian A1 enzymes were studied, with some exhibiting DNA mutator activity on both plasmid and cytoplasmic DNA. Despite this, only mouse A1 was a potent mutator of genomic DNA. These findings show that even if the mouse is devoid of bona fide *A3Z1* gene, mouse A1 can introduce somatic mutations in nuclear DNA, putting the genome at risk of APOBEC fueled oncogenesis.

## Results

### Synthesis and expression of mammalian APOBEC1 sequences

**Fig. 1:**
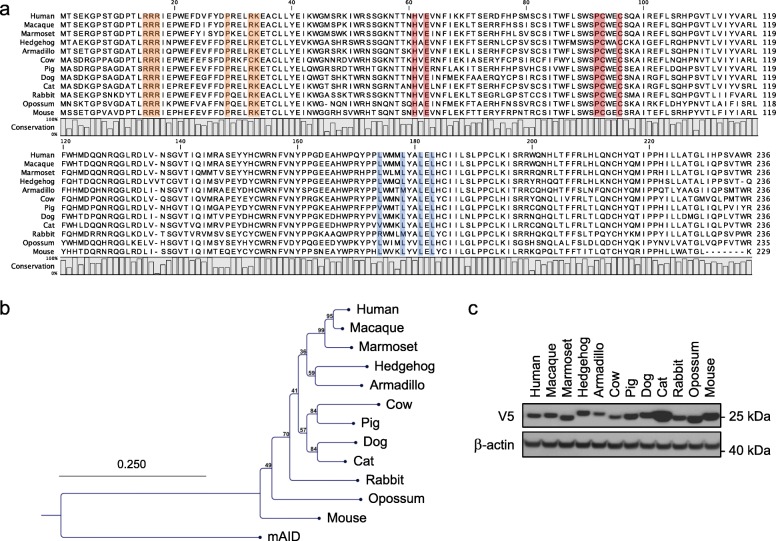
Comparison of APOBEC1 cytidine deaminases. **a** CLUSTALW alignment of A1 protein sequences. Residues involved in zinc coordination are depicted in red. Residues in orange are part of A1 bipartite nuclear localization signal while those involved in nuclear export of A1 are represented in blue. **b** Phylogenetic tree of A1 protein sequences constructed using the Neighbor-joining method with the CLC Main Workbench 7.0.2 software. Mouse AID was used to root the tree. Numbers correspond to bootstrap values inferred from 100,000 replicates. **c** Western blot analysis of V5-tagged A31 proteins in quail QT6 cells. β-actin probing was used as loading control

Mammalian A1 cDNA sequences from several species were retrieved by data mining and synthesized (Fig. 1a, Additional file 1: Table S1). Among them, A1 cDNAs from animals possessing a functional *A3Z1* gene were selected, such as the armadillo, cow, dog, hedgehog, human, macaque, marmoset and rabbit, as well as some from animals known to have lost the *A3Z1* gene during evolution, such as the cat, mouse, pig, and opossum [23, 24, 59]. All harbored the His-X-Glu-X_23–28_-Pro-Cys-X_2–4_-Cys cytidine deaminase domain involved in zinc coordination and enzymatic activity [60] (Fig. 1a, highlighted in red). A phylogenetic analysis of the protein sequences using mouse activation induced deaminase (mAID) as outlier, revealed sub-clustering among mammalian orders *Primates* (human, macaque and marmoset), *Cetartiodactyla* (cow, pig), *Carnivora* (cat, dog) indicating the robustness of the tree (Fig. 1b). Interestingly, the tree suggests that mouse A1 appears to be an outlier to the rest of the A1 sequences.

**Fig. 2:**
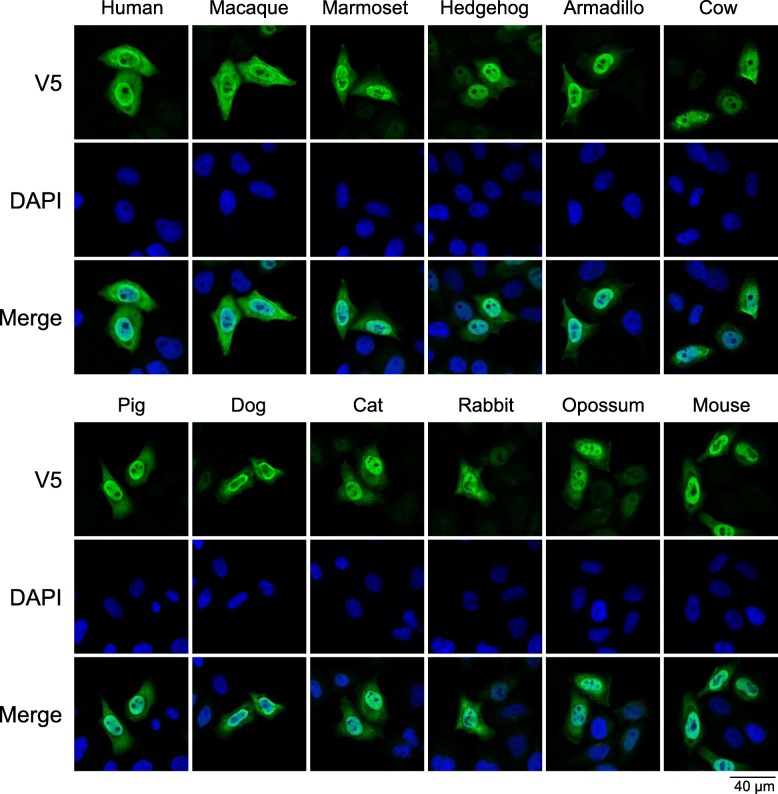
Cellular localization of APOBEC1 cytidine deaminases. Confocal microscopy analysis of V5-tagged A1 proteins in QT6 cells, 24 h post transfection. Nuclei are stained with DAPI

To assess functionality, *A1* cDNAs were cloned in pcDNA3.1 V5-tag encoding expression vector, as well as in a dual promoter vector simultaneously encoding *Bacillus subtilis* phage uracil-DNA glycosylase inhibitor (UGI) gene under a PGK promoter. Expression was then analyzed in quail QT6 cells, as birds are devoid of *APOBEC1* gene and *APOBEC3* locus [61] and are free of any APOBEC editing background [62]. Western-blot analysis reveal that all twelve A1 proteins were expressed with both armadillo A1 and cow A1 being expressed at consistently lower levels compared to the other ten A1s. By contrast the levels of feline A1 were always the highest (Fig. 1c). Confocal microscopy was performed to assess the localization of V5-tagged molecules. All A1 enzymes displayed a nucleocytoplasmic distribution with a strong nuclear localization (Fig. 2). These data are in agreement with A1 nuclear shuttling with the conservation of residues responsible for nuclear addressing (Fig. 1a, orange) and nuclear export (Fig. 1a, blue) [63, 64].

### APOBEC1 DNA cytidine deaminase activity

**Fig. 3:**
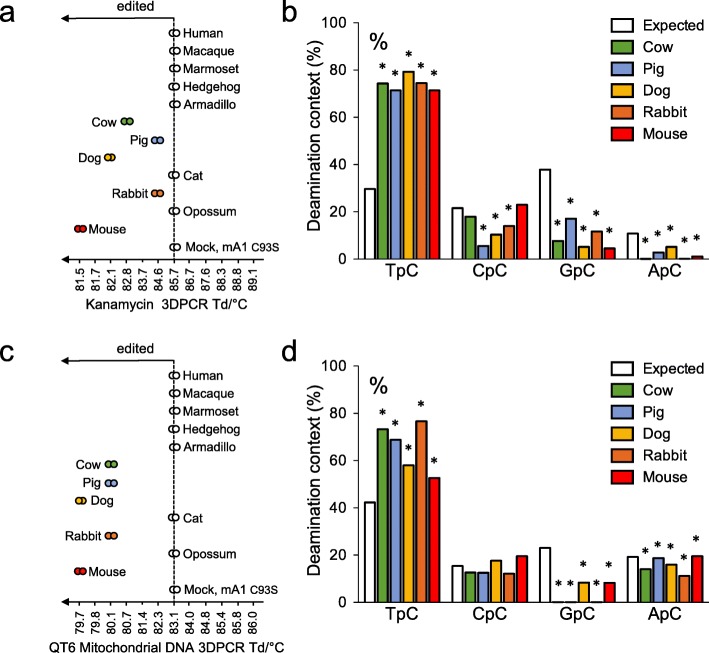
APOBEC1 cytidine deaminase activity on plasmid and cytosolic mitochondrial DNA. **a** Graphical representation of plasmid DNA editing by A1 proteins. The temperature of the DNA products recovered at the lowest Td by *kanamycin* specific 3DPCR amplification are represented on the gradient. **b** Dinucleotide analysis of the deamination context performed on plasmid DNA for PCR products retrieved at 84.6 °C. **c** Graphical representation of *cytochrome c* mtDNA editing by A1 proteins. The last retrieved bands by *cytochrome c* specific 3DPCR amplification are represented on the gradient. **d** Dinucleotide analysis of the deamination context performed on mtDNA for PCR products retrieved at 82.3 °C. Dinucleotide context expected values, based on the dinucleotide composition of DNA sequences are represented by white histograms. * Significant deviation from expected values (χ^2^-test, *P* < 0.05)

To asses A1 enzymatic activity, QT6 cells were transfected with the different *A1* expression plasmids. Total cellular DNA was extracted and DNA editing was assessed on plasmid DNA as well as cytoplasmic mitochondrial DNA, using differential DNA denaturation PCR, 3DPCR. This method exploits the fact that A3-edited DNA is richer in AT, reducing the energy needed to separate DNA strands, allowing PCR amplification of mutated DNA with lower denaturation temperatures compared to reference sequence (Additional file 1: Figure S1). Modulation of the PCR denaturation temperature allows selective amplification of AT-rich DNA, sometimes by up to 10^4^ fold [29]. With primers specific to the kanamycin resistance gene, 3DPCR recovered DNA below the restrictive denaturation temperature of 85.7 °C - obtained with mock plasmid transfection or the mouse A1 catalytic inactive mutant mA1 C93S - for mouse, dog, cow, rabbit and pig A1 constructs with denaturation temperatures between 81.5–84.6 °C (Fig. 3a). To preserve sequence diversity, 3DPCR products obtained at 84.6 °C, just below the restrictive temperature of 85.7 °C were cloned and sequenced. Extensively mutated sequences peppered with C ➔ T and G ➔ A substitutions were identified (Additional file 1: Figure S2A). Dinucleotide context analysis revealed a strong preference for deamination in the 5’TpC dinucleotide context over values “expected” with a random distribution of mutations, where C is the edited base, for all functional A1s (Fig. 3b). This substrate preference for A1s is in keeping with previous work [15, 65]. By analogy with what is known for other APOBEC family members, this deamination preference might be dictated by a previously described hotspot recognition loop present in many polynucleotide cytidine deaminases [66] and may also involve other residues. Similar mutational patterns were obtained using cytoplasmic cytochrome c mitochondrial DNA as target. Once again, only the same five A1 enzymes from mouse, dog, cow, rabbit and pig (Fig. 3c) resulted in editing of target ssDNA. Analysis of 3DPCR products obtained at 82.3 °C again revealed C ➔ T and G ➔ A mutations (Additional file 1: Figure S2B) and a strong preference for the 5’TpC dinucleotide (Fig. 3d). While ssDNA mutator activity has been previously described for both human [11, 16, 19] and opossum A1 enzymes [21] these studies were performed either in *E. coli* or inside hepatitis B virus capsids where the enzyme concentration heavily favors DNA editing [62]. This discrepancy suggests that their activity in a more physiological setting is but modest, and may not edit cytoplasmic DNA sufficiently to be detected by 3DPCR [29].

### APOBEC1 deaminase activity on nuclear DNA

**Fig. 4:**
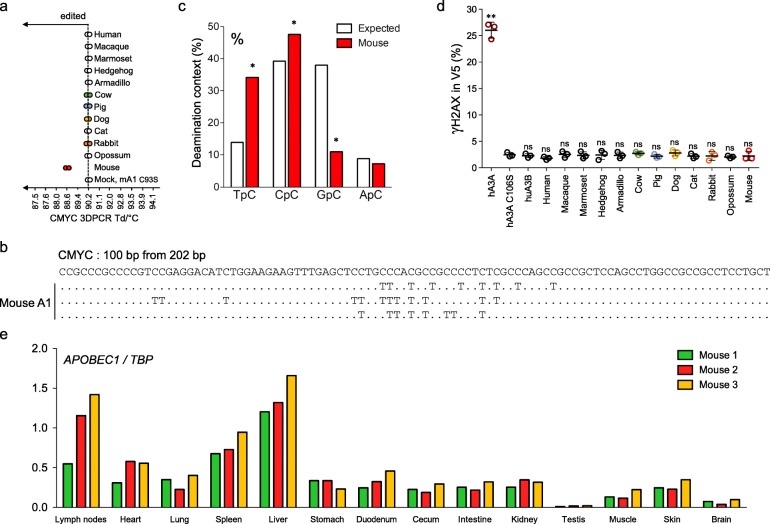
APOBEC1 mediated nuclear DNA editing and damage. **a** Graphical representation of nuclear DNA editing by A1 proteins. The last positive 3DPCR bands retrieved bands by *CMYC* specific 3DPCR amplification are represented on the gradient. **b** Selection of hypermutated *CMYC* sequences after mouse A1-UGI transfection in QT6 cells for PCR products retrieved at 89.4 °C. **c** Dinucleotide analysis of mouse A1 deamination context performed on nuclear DNA for PCR products retrieved at 89.4 °C. Dinucleotide context expected values, based on the dinucleotide composition of DNA sequences are represented by white histograms. * Significant deviation from expected values (χ^2^-test, *P* < 0.05). **d** Double strand breaks formation upon A1 transfection in QT6 cells by flow cytometry analysis of γH2AX staining in V5 transfected cells 48 h post-transfection. Human APOBEC3A (hA3A) was used as positive control. Error bars represent the standard deviations from three independent transfections. Differences compared to human APOBEC3A catalytic mutant hA3A C106S were calculated using student t test (** *p* < 0.01). **e** APOBEC1 expression in 3 C57/BL6 mice tissues normalized on *TBP* reference genes

As all the A1 enzymes displayed a strong nuclear localization (Fig. 2), we next sought to demonstrate whether some of the A1 enzymes could edit chromosomal DNA, a property so far only demonstrated for A3Z1 domain containing APOBEC3 cytidine deaminases typified by APOBEC3A [37, 38, 52, 53, 67]. Accordingly, QT6 cells were co-transfected with plasmids encoding both the *A1* and *UGI* genes from *Bacillus subtilis* to prevent the very efficient removal of uracil bases in nuDNA by UNG that hampers experimental detection of somatic mutations. NuDNA editing was investigated using the 3DPCR technique, that if originally designed to study A3 hyperedited viral genomes can be used to identify sequences with lower mutation frequencies when properly used [68]. Specific 3DPCR amplification of the *CMYC* gene allowed consistent recovery of DNA below the restrictive temperature of Td = 90.2 °C only for mouse A1/UGI transfected cells (Fig. 4a). Molecular cloning and sequencing of PCR products obtained at Td = 89.4 °C confirmed the accumulation of monotonous C ➔ T mutations (Fig. 4b and Additional file 1: Figure S2C), with a deamination preference for 5’TpC and 5’CpC dinucleotide context (Fig. 4c), demonstrating for the first time that mouse A1 can generate somatic mutations in nuclear DNA.

Genomic DNA deamination results in DNA peppered with uracil, that in turn activates base excision repair (BER). Uracil is then removed by UNG and apurinic/apyrimidinic endonucleases cleave the DNA strand for repair or degradation. As a consequence, DSBs can be generated during repair of clustered mutations, when cleavage happens in close proximity on opposite strands [69]. To assess DSB formation following A1 transfection, H2AX histone phosphorylation (γH2AX) in V5 positive cells was quantified by flow cytometry. γH2AX staining of A1 transfected QT6 cells failed to show evidence of DSB formation on a par with the human A3A C106S inactive catalytic mutant. By contrast human A3A (hA3A) expression induced significant DSBs in 25% of hA3A-V5 positive cells (Fig. 4d). To further confirm that DSB formation results from APOBEC mutations processing by UNG, the experiment was repeated by transfecting A3A and mouse A1 expression plasmids co-encoding the UGI UNG inhibitor, abolishing DSB formation (Additional file 1: Figure S3).

This phenotype, somatic mutation in nuclear DNA yet no evidence of DSB formation, is reminiscent of the A3B attenuated activity of human (Fig. 4d) [38, 44], suggesting that both enzymes are not efficient enough to elicited the critical level of mutations triggering DSB formation. One prediction of an attenuated nuclear DNA editing phenotype would be expression in multiple tissues unlike human A3A where basal levels are extremely low [70]. Murine *A1* expression profiles from multiple tissues from 3 mice are given in Fig. 4e. Remarkably, *A1* transcripts were detected in almost every organ tested with a marked expression in liver as well as lymphoid organs such as spleen and lymph nodes (Fig. 4e), independently of the reference gene (*RPL13A*, *TBP* or *HPRT*) used to normalize RTqPCR data (Fig. 4e and Additional file 1: Figure S4). The observation that *A1* is widely expressed is interesting as it suggests that this mutator enzyme is present in many cell types, and could therefore participate to the introduction of somatic mutations in the genome of cells from many tissues.

### Mouse APOBEC1 is the only mouse APOBEC enzyme capable of mutating nuclear DNA

**Fig. 5:**
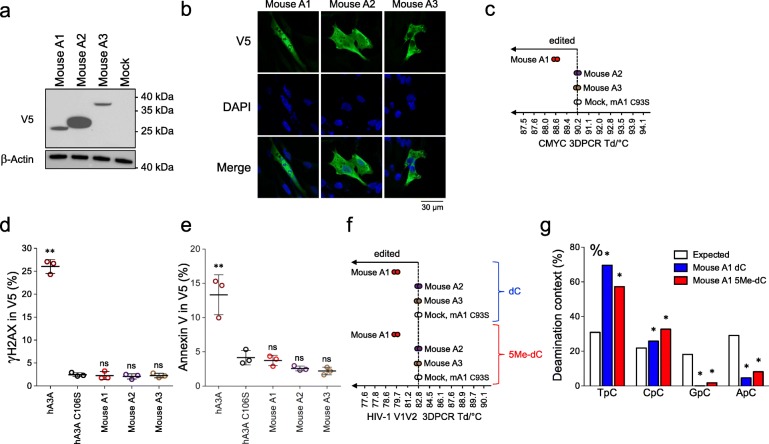
APOBEC1 is the only mouse APOBEC cytidine deaminase capable of mutating nuclear and 5-methylcytidine containing DNA. **a** Western blot analysis of V5-tagged mouse APOBEC cytidine deaminases in quail QT6 cells. β-actin probing was used as loading control. **b** Confocal microscopy analysis of V5-tagged mouse APOBEC cytidine deaminases in QT6 cells, 24 h post transfection. Nuclei are stained with DAPI. **c** Graphical representation of nuclear DNA editing by mouse APOBEC cytidine deaminases. The last retrieved bands by *CMYC* specific 3DPCR amplification are represented on the gradient. **d** Double strand breaks formation upon mouse APOBEC cytidine deaminases transfection in QT6 cells by flow cytometry analysis of γH2AX staining in V5 transfected cells 48 h post-transfection. Human APOBEC3A (hA3A) was used as positive control. Error bars represent the standard deviations of three independent transfections. Differences compared to human APOBEC3A catalytic mutant hA3A C106S were calculated using student t test (** *P* < 0.01). **e** Annexin V staining of apoptosis upon mouse APOBEC cytidine deaminases transfection in HeLa cells by flow cytometry analysis in V5 transfected cells 36 h post-transfection. Differences compared to human APOBEC3A catalytic mutant hA3A C106S were calculated using student t test (** *P* < 0.01). **f** Graphical representation of HIV-1 V1 V2 specific 3D-PCR amplification after QT6 transfections with APOBEC cytidine deaminases plasmids along with a cytidine (dC) or 5-methylcytidine (5Me-dC) containing HIV-1 *env* DNA. **g** Dinucleotide analysis of mouse A1 deamination context performed on HIV-1 V1 V2 sequences obtained at 81.2 °C from DNA containing either cytidine (dC) or 5-methylcytidine (5Me-dC). Dinucleotide context expected values, based on the dinucleotide composition of DNA sequences are represented by white histograms. * Significant deviation from expected values (χ^2^-test, *P* < 0.05)

To date, mouse APOBEC2 (A2) is devoid of catalytic activity while mouse APOBEC3 (A3) can restrict some retroviruses [15] and edit cytoplasmic mitochondrial DNA [37]. When overexpressed mouse A2 displayed a classical nucleocytoplasmic distribution while A3 was strictly cytoplasmic (Fig. 5a, b). However, only mouse A1 was able to introduce somatic mutations in nuclear DNA using *CMYC* specific 3DPCR (Fig. 5c). In keeping with the lack of cytidine deaminase activity on nuclear DNA, mouse A2 and A3 both failed to elicit DSBs or apoptosis following transfection, just like mouse A1 (Figs. 5d, e).

### Mouse APOBEC1 can deaminate 5-methylcytidine containing ssDNA

To date, only A3 Z1 domain enzymes that edit chromosomal DNA also deaminate 5-methylcytidine residues on ssDNA [38, 52, 53, 57]. As one report demonstrates an in vitro 5Me-dC deamination activity of an oligonucleotide by mouse A1 [56] we explored 5Me-dC deamination *in cellulo* using a protocol previously described for human A3A and A3B [38, 57]. Fully 5Me-dC substituted PCR fragments were made and transfected into QT6 cells. 3DPCR recovered DNA down to Td = 79.7 °C, with mouse A1 transfection, below the restrictive denaturation temperature of Td = 82.8 °C, while mouse A2 and A3 both failed to edit either 5’TpC or 5’Tp5MedC DNA (Fig. 5f). Sequencing of cloned products revealed CG ➔ TA hypermutations (Additional file 1: Figure S2D) with a strong 5’TpC / 5’Tp5MedC deamination bias after A1 transfection (Fig. 5g). As 5Me-dC deamination results in thymidine, which is processed by mismatch repair mechanisms far less efficient than one involving uracil removal by UNG, 5Me-dC deamination by mouse A1 could contribute to the numerous 5MeCpG deamination hotspots evidenced in many genes associated with cancer [39, 71]. On top of that 5Me-dC deamination could be involved in removing epigenetic marks [72], with documented consequences in cancer formation [73].

## Discussion

The data presented here indicates that among all 12 APOBEC1 enzymes tested, only five - cow, pig, dog, rabbit and mouse - were found to exhibit DNA mutator activity, introducing hypermutations in several DNA targets in vivo. Among them, opossum A1, pig A1 and mouse A1 originate from species devoid of a functional APOBEC3 Z1 cytidine deaminase, known to put the nuclear genome at risk of somatic mutations. Further analysis revealed that among all the A1 tested, mouse A1 singularly displayed a nuclear DNA mutator activity associated with deamination of 5Me-dC containing DNA which was up to now a hallmark of APOBEC3 Z1 catalytic domain [38, 51, 57].

However, if mouse A1 consistently edited nuclear DNA, its activity appears to be moderate, failing to generate DSBs. In this respect, it is similar to the hypomutator phenotype of its human A3B counterpart [38, 44]. Unlike human A3B, mouse A1 expression doesn’t result in apoptosis [38] (Fig. 5e), further indicating that its mutagenic activity is modest. However, this hypomutator phenotype should not be underestimated as a source of somatic mutations in cancer formation as it is suggested that mismatch repair machinery efficiency is limited to several hundred mutations in a single event [74]. If only few genomics studies of murine cancers have been performed, it appears that the dominant mutations are CG ➔ TA transitions [75], some of them presenting the characteristic mutational signatures 2 and 13 associated with APOBEC3 deamination [76]. Noteworthy, mice harboring *A1*^−/−^ deficiency present a decreased gastro-intestinal tumor burden [77], further stressing the putative link between mouse A1 expression and cancer onset.

If in our study only mouse A1 was demonstrated to induce hypermutation in nuDNA, one cannot exclude that other A1 may also induce mutations in chromosomal DNA, albeit below the experimental detection of 3DPCR threshold which is in the order of 2–4 substitutions per kb^− 1^ [29, 68]. Indeed, a growing number of studies also points to human A1 expression being associated with GC ➔ TA somatic mutations peppering many cancer genomes. A strong association between human APOBEC1 expression and the APOBEC mutational signature was found in esophageal adenocarcinomas [78] and APOBEC1 expression was also correlated with indel mutations in many tumor genomes [79]. Moreover, a fine analysis of mutational footprints was able to extract a specific APOBEC1 mutational motif that can be found in many human cancer genomes [80]. Similarly, although rabbit A1 was found inactive on nuclear DNA in our experimental setup, over-expression of rabbit A1 in transgenic animals results in hepatocellular carcinoma [58], suggesting that the enzyme may under some conditions contribute to tumorigenesis. Thus, the same can be true for other A1 deaminases in vivo, when the complex and poorly understood regulation of cytidine deaminase activity fails. Future genomic analyses of mammalian cancer genomes will certainly help unravel signatures and shed light on the etiological agents [41, 81].

## Conclusions

At an experimental level mouse APOBEC1 is remarkable among 12 mammalian A1 enzymes in that it represents a source of somatic mutations in mouse genome, potentially fueling oncogenesis. While the *Rodentia* order is bereft of A3A and A3B like enzymes it seems that APOBEC1 may well substitute for it, albeit remaining much less active. This modifies the paradigm that APOBEC3 and AID enzymes are the sole endogenous mutator enzymes giving rise to off-target editing of mammalian genomes.

## Methods

### Plasmids

Mammalian APOBEC1 cDNAs, from armadillo, cat, cow, dog, hedgehog, human, macaque, marmoset, mouse, opossum, pig and rabbit were synthesized (GeneCust), amplified by PCR and cloned into pcDNA3.1D/V5-His-TOPO vector (Life Technologies) (Additional file 1: Table S1). Mouse A1 C93S inactive catalytic mutant was obtained by site directed mutagenesis using standard protocol (GeneArt Site-Directed Mutagenesis System, Life Technologies) (Additional file 1: Table S2). Human APOBEC3A and APOBEC3A C106S, mouse APOBEC2 and mouse APOBEC3 plasmids were previously described [15, 37]. Dual promoter vector encoding uracil-DNA glycosylase inhibitor UGI from *Bacillus subtilis* phage, was generated using BamHI/NheI restriction sites to substitute PGK driven GFP sequence from pSF-CMV-PGK-daGFP vector (Sigma) by UGI sequence cloned into pcDNA3.1 vector. APOBEC1 coding sequences were cut from pcDNA3.1D/V5-His-TOPO vectors using HindIII and PmeI and cloned into pSF-CMV-PGK-UGI using HindIII and EcoRV restriction sites. All constructs were grown in *E. coli* TOP10 cells (Life Technologies) and verified by sequencing.

### Cell lines

Japanese quail embryonic fibroblast QT6 cells (ATCC CRL 1708) were obtained commercially from LGC STANDARDS and maintained in Ham’s medium supplemented with 1% chicken serum, 10% fetal bovine serum, 5% tryptose phosphate, 2 mM L-glutamine, 50 U/ml penicillin and 50 mg/ml streptomycin. Human HeLa cells (ATCC CCL2) were obtained commercially from LGC STANDARDS and were maintained in DMEM glutamax medium (Life Technologies) supplemented with 10% FCS, 50 U/ml penicillin and 50 mg/ml streptomycin.

### Transfections

Plasmid transfections were performed with 2 μg of DNA for 8 × 10^5^ of QT6 cells using Fugene HD (Promega) and harvested after 48 h. For immunofluorescence labeling, 5 × 10^4^ cells grown on chamber slides (LabTek) were transfected with 1 μg of expression plasmids using Fugene HD (Promega) following manufacturer’s recommendations.

### Western blotting

Transfected cells were resuspended in lysis buffer (0.5% Nonidet P-40, 20 mM Tris-HCl pH 7.4, 120 mM NaCl and 1 mM EDTA) supplemented with Complete Protease Inhibitor Mixture (Roche Applied Science). Cell lysates were clarified by centrifugation at 14,000×g for 10 min and Western blot analysis on cell lysates was carried out as previously described [38].

### Immunofluorescence

After PBS washings, transfected cells grown on chamber slides were fixed and permeabilized, and immunofluorescence V5 staining was performed as previously described [44].

### FACS analysis of double strand breaks

At 48 h after transfection, FACS analysis of double strand breaks in V5 positive cells was performed using γH2AX staining as described in [44].

### DNA extraction and 3DPCR amplification

Total DNA from transfected cells was extracted, all PCR amplification were performed as previously described [38] with the cycling conditions and primers are presented in Additional file 1: Table S3. PCR products were cloned into TOPO 2.1 vector (Life Technologies) and sequencing outsourced to Eurofins. Expected values are derived from the base composition of the target sequence assuming no dinucleotide bias (% of NpC = numbers of NpC/numbers of Cs) × 100).

### RNA extraction and real time PCR amplification

C57BL/6 Mouse tissues were incubated in RNA later stabilization reagent, and mechanically disrupted before extraction of total RNA using RNeasy® lipid tissue mini kit (Qiagen) according to the manufacturer’s protocol. Corresponding cDNAs were synthetized using QuantiTect reverse transcription kit (Qiagen). Quantification was performed by TaqMan using Takyon Rox probe mastermix dTTP blue (Eurogentec). Sequences of specific primers and probes used are detailed in Additional file 1: Table S4. Cycling conditions were as follows: first step of denaturation at 95 °C during 10 min. Followed by 40 cycles of amplification (95 °C 15 s., 58 °C 15 s. and 68 °C 15 s.). Fluorescence was measured during the 68 °C step incubation using a Realplex2 Mastercycler (Eppendorf). The specificity of the PCR products was verified by sequencing. Messenger RNA expression levels were normalized based on the *RPL13A*, *TBP* and *HPRT* reporter genes.

### Flow-cytometry analysis of apoptosis

Transfected HeLa cells were harvested, incubated at 37 °C in DMEM complete medium, for 30 min. After PBS washings, cells were resuspended in binding buffer and stained with Annexin-eFluor 450 following Annexin V Apoptosis Detection Kit eFluor™ (ThermoFischer) standard protocol. After fixation in 2% ice-cold paraformaldehyde (Electron Microscopy Sciences) for 10 min and permeabilization in 90% ice-cold methanol (Sigma) for 30 min, cells were incubated 1 hour with 1:100 diluted Alexa Fluor 488-conjugated mouse monoclonal anti-V5 antibody (AbD Serotec) on ice. After PBS washings stained samples were acquired on a MACSQuant Analyser (Miltenyi Biotech). Data were analyzed with FlowJo software (Tree Star Inc. version 8.7.1).

## Additional file

**Additional file 1: Figure S1.** Differential DNA denaturation 3DPCR. **A)** APOBEC cytidine deaminases deaminate cytidine into uridine in single stranded DNA. **B)** APOBEC activity leads to the. Accumulation of GC à AT mutations. **C)** As GC basepairs with 3 hydrogen bonds and AT with 2 hydrogen bonds, AT rich DNA. requiers less energy for denaturation allowing PCR amplification at lower denaturation Td/°C **D)** PCR amplification with a gradient. of denaturation temperatures allows to pickup AT rich APOBEC mutated DNA below the restrictive temperature of non mutated. DNA, represented by the yellow dotted line. **Figure S2.** Mutation matrices of APOBEC1 mutated sequences. **Figure S3.** Double strand breaks formation upon APOBEC transfection requires UNG. Double strand breaks formation upon A1 transfection in QT6 cells by flow cytometry analysis of γH2AX staining in V5 transfected cells 48. hours post-transfection. Human APOBEC3A (hA3A) was used as positive control. Circles represent data from γH2AX staining upon.transfection with pcDNA3.1 APOBEC plasmids while squares represent γH2AX staining upon transfection with a dual promoter vector coexpressing. APOBEC sequences along with the UGI UNG inhibitor. Error bars represent the standard deviations from three independent transfections. Differences between pcDNA3.1 and pSF-UGI transfections were calculated using student t test (** *p* < 0.01). **Figure S4.** Expression profile of APOBEC1. APOBEC1 expression in 3 C57/BL6 mice tissues normalized on RPL13A, TBP, and HPRT reference genes. **Table S1.** Compendium of primers used for APOBEC1 amplification and cloning. **Table S2.** Primers used for mutagenesis. **Table S3.** Compendium of primers and PCR conditions used for Nested PCR/3DPCR amplifications. **Table S4.** Compendium of primers and UPL probes used for mouse transcriptome analysis.

## Data Availability

Data sharing is not applicable to this article as no data libraries were generated. Accession numbers for the various APOBEC sequences are available in Additional file 1: Table S1. Sequences obtained after 3DPCR amplification, that were used in the present manuscript are available in fasta format in Additional file 1. The communication author will accommodate requests of relevant materials.

## Abbreviations

3DPCR: differential DNA denaturation PCR
5Me-dC: 5-methylcytidine
A1: APOBEC1
A3A: APOBEC3A
A3B: APOBEC3B
ACF: APOBEC1 complementation factor ACF
APOBEC: apolipoprotein B mRNA Editing Catalytic Polypeptide-like
BER: base excision repair
CBX6: chromobox 6
CBX7: chromobox 7
cDNA: complementary DNA
DNA: deoxyribonucleic acid
DSB: double strand break
GFP: green fluorescent protein
HPRT: hypoxanthine Phosphoribosyltransferase
Kb: kilo base
mAID: mouse activation induced deaminase
mtDNA: mitochondrial DNA
nuDNA: nuclear DNA
PCR: polymerase chain reaction
PGK: phosphoglycerate kinase
RNA: ribonucleic acid
RPL13A: Ribosomal Protein L13a
RTqPCR: reverse transcription quantitative PCR
ssDNA: single stranded DNA
TBP: TATA binding protein
Td: denaturation temperature
UGI: uracil-DNA glycosylase inhibitor
UNG: uracil-DNA glycosylase
γH2AX: Phosphorylated histone H2AX
