# Supplementary material for: Mouse APOBEC1 cytidine deaminase can induce somatic mutations in chromosomal DNA
Source: BMC Genomics. 2019 Nov 14;20:858. doi: 10.1186/s12864-019-6216-x (PMC6854741; doi:10.1186/s12864-019-6216-x)
Supplement: Supplementary file 1 — Additional file 1: Figure S1. Differential DNA denaturation 3DPCR. A) APOBEC cytidine deaminases deaminate cytidine into uridine in single stranded DNA. B) APOBEC activity leads to the. Accumulation of GC à AT mutations. C) As GC basepairs with 3 hydrogen bonds and AT with 2 hydrogen bonds, AT rich DNA. requiers less energy for denaturation allowing PCR amplification at lower denaturation Td/°C D) PCR amplification with a gradient. of denaturation temperatures allows to pickup AT rich APOBEC mutated DNA below the restrictive temperature of non mutated. DNA, represented by the yellow dotted line. Figure S2. Mutation matrices of APOBEC1 mutated sequences. Figure S3. Double strand breaks formation upon APOBEC transfection requires UNG. Double strand breaks formation upon A1 transfection in QT6 cells by flow cytometry analysis of γH2AX staining in V5 transfected cells 48. hours post-transfection. Human APOBEC3A (hA3A) was used as positive control. Circles represent data from γH2AX staining upon.transfection with pcDNA3.1 APOBEC plasmids while squares represent γH2AX staining upon transfection with a dual promoter vector coexpressing. APOBEC sequences along with the UGI UNG inhibitor. Error bars represent the standard deviations from three independent transfections. Differences between pcDNA3.1 and pSF-UGI transfections were calculated using student t test (** p < 0.01). Figure S4. Expression profile of APOBEC1. APOBEC1 expression in 3 C57/BL6 mice tissues normalized on RPL13A, TBP, and HPRT reference genes. Table S1. Compendium of primers used for APOBEC1 amplification and cloning. Table S2. Primers used for mutagenesis. Table S3. Compendium of primers and PCR conditions used for Nested PCR/3DPCR amplifications. Table S4. Compendium of primers and UPL probes used for mouse transcriptome analysis. [file 12864_2019_6216_MOESM1_ESM.pdf]

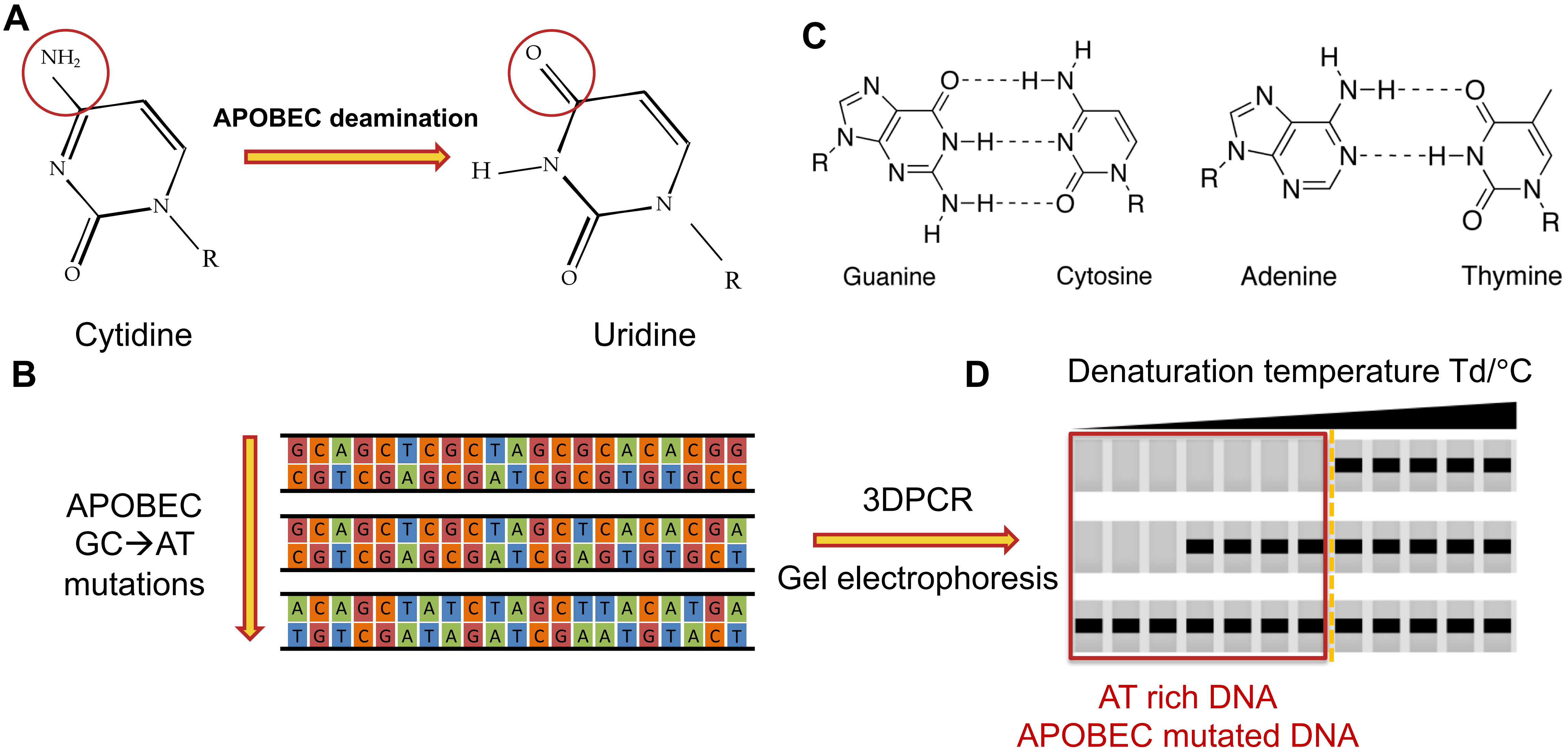

Supplementary Figure 1 : Differential DNA denaturation 3DPCR.
A) APOBEC cytidine deaminases deaminate cytidine into uridine in single stranded DNA. **B)** APOBEC activity leads to the accumulation of GC → AT mutations. **C)** As GC basepairs with 3 hydrogen bonds and AT with 2 hydrogen bonds , AT rich DNA requiers less energy for denaturation allowing PCR amplification at lower denaturation Td/°C **D)** PCR amplification with a gradient of denaturation temperatures allows to pickup AT rich APOBEC mutated DNA below the restrictive temperature of non mutated DNA, represented by the yellow dotted line.

A

Kanamycin 3D-PCR at 84.6°C

Mouse

↱	T	C	G	A
T		1	0	0
C	59		0	1
G	0	0		87
A	0	0	0	

n=2430 bp

Rabbit

↱	T	C	G	A
T		0	0	0
C	53		1	0
G	0	0		43
A	0	0	1	

n=2565 bp

Pig

↱	T	C	G	A
T		0	0	0
C	42		0	0
G	0	0		35
A	1	0	0	

n=2430 bp

Cow

↱	T	C	G	A
T		0	0	0
C	93		0	0
G	0	0		39
A	0	0	0	

n=2565 bp

Dog

↱	T	C	G	A
T		0	0	0
C	63		0	0
G	0	0		58
A	0	0	1	

n=2160 bp

B

Cytochrome C 3D-PCR at 82.3°C

Mouse

↱	T	C	G	A
T		0	1	0
C	0		0	0
G	0	0		133
A	0	0	1	

n=1288 bp

Rabbit

↱	T	C	G	A
T		0	0	0
C	0		0	0
G	0	0		107
A	0	0	0	

n=1656 bp

Pig

↱	T	C	G	A
T		0	1	0
C	33		0	0
G	0	0		16
A	1	0	0	

n=920 bp

Cow

↱	T	C	G	A
T		0	0	0
C	0		0	0
G	0	0		71
A	0	0	0	

n= 1472 bp

Dog

↱	T	C	G	A
T		1	0	0
C	0		0	1
G	0	0		117
A	0	0	1	

n=1288 bp

C

CMYC 3D-PCR at 89.4°C

Mouse

↱	T	C	G	A
T		0	0	0
C	82		0	0
G	0	0		0
A	0	0	0	

n=1616 bp

D

HIV-1 V1V2 3D-PCR at 81.2°C

Mouse dC

↱	T	C	G	A
T		1	0	1
C	5		0	0
G	0	0		283
A	1	0	1	

n=3948 bp

Mouse 5MedC

↱	T	C	G	A
T		1	0	2
C	24		0	1
G	0	0		110
A	0	0	1	

n=2820 bp

Supplementary Figure 2 : Mutation matrices of APOBEC1 mutated sequences.

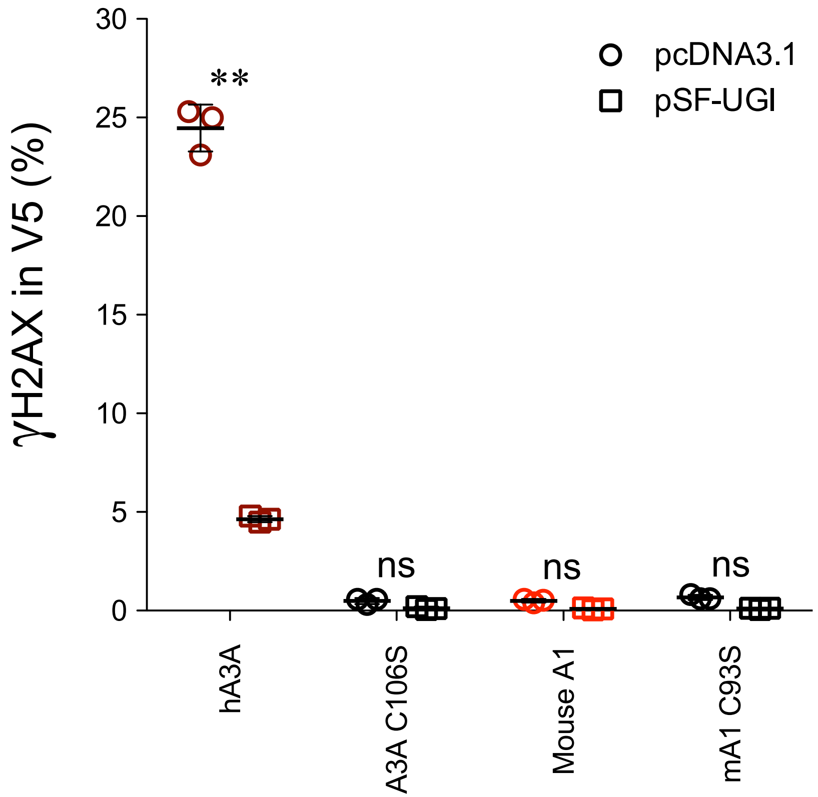

Supplementary Figure 3 : Double strand breaks formation upon APOBEC transfection requires UNG.

Double strand breaks formation upon A1 transfection in QT6 cells by flow cytometry analysis of γH2AX staining in V5 transfected cells 48 hours post-transfection. Human APOBEC3A (hA3A) was used as positive control. Circles represent data from γH2AX staining upon transfection with pcDNA3.1 APOBEC plasmids while squares represent γH2AX staining upon transfection with a dual promoter vector co-expressing APOBEC sequences along with the UGI UNG inhibitor. Error bars represent the standard deviations from three independent transfections. Differences between pcDNA3.1 and pSF-UGI transfections were calculated using student t test (** $p<0.01$).

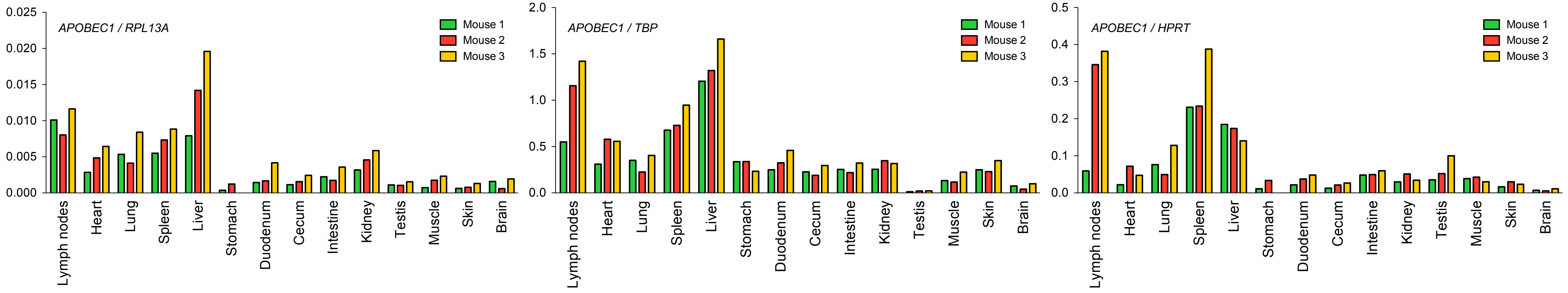

Supplementary Figure 4 : Expression profile of APOBEC1.
APOBEC1 expression in 3 C57/BL6 mice tissues normalized on RPL13A, TBP, and HPRT reference genes.

Plasmid	Matrix	Primers
Human A1	NM_001644	For : 5'- CACC**ATG**ACTTCTGAGAAAGGTCC<br>Rev : 5'- TCTCCAAGCCACAGAAGGATGTATC
Rabbit A1	NM_001082341	For : 5'- CACC**ATG**GCTTCCGAGAAAGGTC<br>Rev : 5'- TCTCCAAGGCACAGAAGGTTG
Mouse A1	NM_031159	For : 5'- CACC**ATG**AGTTCCGAGACAGGCC<br>Rev : 5'- TTTCAACCCTGTAGCCCAAAGG
Opossum A1	NM_001032982	For : 5'- CACC**ATG**AATTCTAAGACAGGTCCA<br>Rev : 5'- TCTCCAGGTCACAAATGGCTGG
Armadillo A1	XM_004455274	For : 5'- CACC**ATG**ACTTCTGAGACAGGTCCTTC<br>Rev : 5'- TCTCCAAGTCATGGAAGGTTGTATT
Cow A1	XM_002687817	For : 5'- CACC**ATG**GCTTCTGACAGAGGTCCTCCA<br>Rev : 5'- TCTCCAAGTCATAGGAAGTTGTACC
Pig A1	XM_003126519	For : 5'- CACC**ATG**GCTTCTGACAGAGGTCCTTC<br>Rev : 5'- TCTGTAAATCACAGGTAGTTGTATC
Macaque A1	XM_005570020	For : 5'- CACC**ATG**ACTTCTGAGAAAGGTCCTTC<br>Rev : 5'- TCTCCAAGTCACGGAAGGCTGTATC
Marmoset A1	XM_009003530	For : 5'- ATGACTTCTGAGAGAGGTCCTTCC<br>Rev : 5'- TCTCCAAGTCACAGAAGGCTGGATC
Hedgehog A1	XM_004717433	For : 5'- CACC**ATG**ACTGCTGAGAAAGGTCCTTC<br>Rev : 5'- CTGAGTCACAGAAGGTGGTATCAAC
Dog A1	XM_543826	For : 5'- CACC**ATG**GCTTCTGACAAAGGTCCTTC<br>Rev : 5'- TCTCCAAGTCACAAGAGGTTGTATC
Cat A1	XM_543826	For : 5'- CACC**ATG**GCTTCTGACAAAGGTCCTTC<br>Rev : 5'- TCTCCAAGTCACAAGAGGTTGTATC

Supplementary table 1 : Compendium of primers used for APOBEC1 amplification and cloning

Plasmid	Matrix	Primers
Mouse A1 C93S	pcDNA3.1<br>Mouse A1	For : 5’- CTGTCCTGGAGTCCC**AGC**GGGGAGTGCTCCAGG<br>Rev : 5’- CCTGGAGCACTCCCC**GCT**GGGACTCCAGGACAG

Supplementary table 2 : Primers used for mutagenesis

Primer	Application	Sequence	Cycling conditions
Kanamycin out fwd	kanamycin PCR	5’- CGACCTGTCCGGTGCCCTGAATGAA	95°C 5 min<br>37x (95°C 30 sec, 60°C 30 sec, 72°C 1 min), 72°C 10 min
Kanamycin out rev	kanamycin PCR	5’- GACGGCCACAGTCGATGAATCCAGAA	
Kanamycin in fwd	kanamycin 3DPCR	5’- TCATCTCACCTTGCTCCTGCCGAGAA	81-89°C 5 min<br>42x (81-89°C 1 min, 60°C 45 sec, 72°C 2 min), 72°C 10 min
Kanamycin in rev	kanamycin 3DPCR	5’- CTTCGTCCAGATCATCCTGATCGACAA	
CytC out fwd	Cytochrome C PCR	5’- CATCCAAAGCAGGTCAACCTATGACAA	95°C 5 min<br>35x (95°C 30 sec, 60°C 30 sec, 72°C 1 min), 72°C 10 min
CytC out rev	Cytochrome C PCR	5’- TCTTGGTTAAGAGGTGTGGTCTGTAA	
CytC in fwd	Cytochrome C 3DPCR	5’- CCTCTACACAGCGTAGACTTACATCAA	80-86°C 5 min<br>35x (80-86°C 1 min, 60°C 30 sec, 72°C 2 min, 72°C 10 min)
CytC in rev	Cytochrome C 3DPCR	5’- AAGACCTCGTTTAGCCATTCATACAA	
QT6 cMyc out fwd	cMyc PCR	5’- GATGCCGCTCAGCGCCAGCCTCCCCAGCAA	95°C 5 min<br>40x (95°C 1 min, 58°C 45 sec, 72°C 2 min), 72°C 10 min
QT6 cMyc out rev	cMyc PCR	5’- GCAGTCCTGGATGATGATGGATTTGACGAA	
QT6 cMyc in fwd	cMyc 3DPCR	5’- TTCGAGGAGGAGGAGGAGAACTTCTA	87-95°C 5 min<br>42x (87-95°C 1 min, 60°C 1 min, 72°C 2 min), 72°C 10 min
QT6 cMyc in rev	cMyc 3DPCR	5’- GATTCGTCGTCCGGGTCGCAGATGAA	

Supplementary Table 3 : Compendium of primers and PCR conditions used for nested PCR/3DPCR amplifications

**Target**	**UPL Probe**	**Primers**
Mouse APOBEC1	27	For : 5’-CCCATGAGCGTTGGATTC<br>Rev : 5’-TCAACCACGGGCAGTCTT
Mouse ribosomal protein L13A (RPL13A)	108	For : 5’- CCCTCCACCCTATGACAAGA<br>Rev : 5’- GCCCCAGGTAAGCAAACTT
Mouse hypoxanthine guanine phosphoribosyl transferase (HPRT)	95	For : 5’- TCCTCCTCAGACCGCTTTT<br>Rev : 5’- CCTGGTTCATCATCGCTAATC
Mouse TATA box binding protein (TBP)	107	For : 5’- GGCGGTTTGGCTAGGTTT<br>Rev : 5’- GGGTTATCTTCACACACCATGA

Supplementary Table 4 : Compendium of primers and UPL probes used for mouse transcriptome analysis.
